# Highly Sensitive Detection of Naphthalene in Solvent Vapor Using a Functionalized PBG Refractive Index Sensor

**DOI:** 10.3390/s120202018

**Published:** 2012-02-13

**Authors:** Maiko Girschikofsky, Manuel Rosenberger, Stefan Belle, Malte Brutschy, Siegfried R. Waldvogel, Ralf Hellmann

**Affiliations:** 1 University of Applied Sciences Aschaffenburg, Würzburger Straße 45, D-63743 Aschaffenburg, Germany; E-Mails: manuel.rosenberger@h-ab.de (M.R.); stefan.belle@h-ab.de (S.B.); ralf.hellmann@h-ab.de (R.H.); 2 Institute of Organic Chemistry, Johannes Gutenberg-University Mainz, Duesbergweg 10-14, D-55128 Mainz, Germany; E-Mails: brutschy@uni-mainz.de (M.B.); waldvogel@uni-mainz.de (S.R.W.)

**Keywords:** gas detection, optical sensor, Bragg grating, cyclodextrin, aromatic hydrocarbons

## Abstract

We report an optical refractive index sensor system based on a planar Bragg grating which is functionalized by substituted *γ*-cyclodextrin to determine low concentrations of naphthalene in solvent vapor. The sensor system exhibits a quasi-instantaneous shift of the Bragg wavelength and is therefore capable for online detection. The overall shift of the Bragg wavelength reveals a linear relationship to the analyte concentration with a gradient of 12.5 ± 1.5 pm/ppm. Due to the spectral resolution and repeatability of the interrogation system, this corresponds to acquisition steps of 80 ppb. Taking into account the experimentally detected signal noise a minimum detection limit of 0.48 ± 0.05 ppm is deduced.

## 1. Introduction

Within the last two decades optical fiber-based methods for bio- and chemosensing have gained considerable and continuously growing interest. In addition to a fast and secure data transmission such devices are characterized by a very compact, highly sensitive type of construction. However, the major advantages for the application in the field of gas sensing are the possibility of remote monitoring and the non-emission and non-sensitivity to electromagnetic fields which recommends their usage in potentially explosive atmospheres.

The most commonly reported sensing techniques are either on labeled or direct optical detection, whereat the label-free variation is principally based on either remission, refractivity, interference or a combination of those principles [1]. Several technology platforms such as the surface plasmon resonance [2], the ring resonator [3], interferometers [4,5] as well as several arrangements of waveguide based systems [6,7] take advantage of the refractivity, which describes the dependence on the refractive index of a system.

The refractive index sensor used in this study is based on a planar waveguide including a planar Bragg grating (PBG). Bragg gratings act as a reflector for a particular wavelength (referred to as the Bragg wavelength *λ_B_*), which is achieved by a periodic perturbation of the refractive index in the waveguide core. An unilateral disclosure of the Bragg grating provides the penetration of the evanescent field into the medium surrounding the sensor. The penetration depth is, in general, on the order of or less than the wavelength of the guided light [8]. Hence, changes in the refractive index of the medium lead to a certain change of the effective refractive index and consequently the wavelength of the reflected light. The sensor was already used in previous works for monitoring DNA hybridization [9] and the composition of alternative fuels such as biodiesel [10].

To demonstrate the high sensitivity of the sensor system for the detection of aromatic hydrocarbons, we have chosen naphthalene since it has a comparatively low vapor pressure corresponding to a low volume ratio of the constituent in the saturated gas phase. Naphthalene is a colorless crystalline compound and primarily used as insecticide, solvent or in explosive applications [11]. The functionalization of the sensor against naphthalene was performed by dip coating the sensor surface with a substituted *γ*-cyclodextrin.

## 2. Experimental

### 2.1. Sensor Chip

The sensor used in these studies consists of three silica layers upon a silicon wafer providing a planar multimode waveguide. For the realization of Bragg gratings, periodic perturbations of the refractive index were written into the core using direct UV writing method. To ensure evanescent field interaction of the guided mode, a subsequent hydrofluoric etching step opens a patterned window by partly removing the upper cladding layer leading the PBG to act as a refractive index sensor. In order to regard mutilation by temperature fluctuations, a second grating is written into the waveguide apart from the opened sensing window, thus it is not influenced by any changes of the refractive index and serves as a reference to account any temperature effect. Further details on the manufacturing process and the topography as measured by a confocal laser scanning microscope have been published by the authors and others elsewhere [12–15].

**Figure 1.**
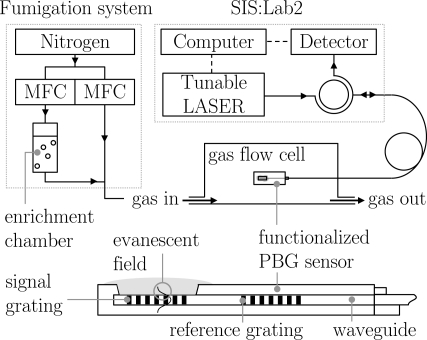
Schematic representation of the experimental setup with an additional longitudinal cross section of the sensor.

A basic illustration of the experimental setup with an additional longitudinal cross section of the PBG sensor is shown in Figure 1.

### 2.2. Materials

#### Cyclodextrin

Cyclodextrin are cyclic oligosaccharides containing six (*α*), seven (*β*) or eight (*γ*) D-glucopyranoside units linked by a 1,4-glycosidic bond. The exterior of their cone-shaped structure is hydrophilic whereas the cavity is hydrophobic, which favors the accommodation of an organic molecule of appropriate dimensions leading to a non-covalent inclusion complex [16]. Through modification, native cyclodextrin become effective templates for the generation of a wide range of molecular hosts [17,18]. The employed octakis(2,3,6-tri-*O*-allyl)-*γ*-cyclodextrin was synthesized by the method of Leydet *et al*. treating *γ*-cyclodextrin with an excess of sodium hydroxide and allylchloride in dimethyl sulfoxide at room temperature [19]. The perallylated product is a highly viscous material. The complete allylation was confirmed by ^1^H NMR spectroscopy. This particular affinity material has found previous application in the gravimetric sensing of peroxidic explosives [20].

#### Naphthalene

Naphthalene (an organic compound with the formula C_10_H_8_) belongs to the group of potentially carcinogenic arenes and consists of two benzene rings fused together referred to as benzenoid polycyclic aromatic hydrocarbon. The analytically pure naphthalene was employed as purchased.

### 2.3. Instrumentation

#### Fumigation system

The fumigation system is based on the principle of dynamic saturation method, in which a complementary gas is brought into contact with the condensed phase of a constituent at a certain temperature to ensure saturation condition. The complementary gas used in this study is nitrogen, which is fully saturated with naphthalene at a temperature of 20 °C to achieve a concentration of 80 ppm v/v [21]. By diluting the saturated nitrogen with pure nitrogen using mass flow controllers (MFC), a defined final concentration is reached and applied to the sensor [22,23].

#### Interrogation system

The used interrogation system by Stratophase Ltd. (SIS:Lab2) covers the spectral region of 1510 nm – 1590 nm by a tunable laser source and has a spectral resolution of 1 pm at a refresh rate of 2 Hz. Through multiple peak detection and tracking, the system permits monitoring the shift of the reflected wavelength λ*_B_* and therefore the time-based response of the sensor system.

### 2.4. Sensor Functionalization

**Figure 2.**
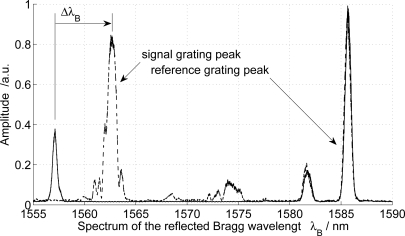
Spectrum of the reflected Bragg wavelength before (—–) and after (- - -) functionalization.

For functionalization, the cyclodextrin derivative was dissolved in tetrahydrofuran in composition of 50 mg/mL and applied to the sensor using the dip coating system by MTI Coporation. The coating was performed at room temperature with an entry and withdrawal speed of 100 mm/min and a dwell time of approximately 10 s. The functionalization was confirmed by a significant spectral shift of the simultaneously monitored reflected Bragg wavelength (Figure 2) induced by the increased effective refractive index.

Figure 2 also reveals a significant increase of the reflected intensity which can be attributed to an increase of the effective refractive index as shown in [24].

### 2.5. Target Molecule Application and Detection

The functionalized sensor was exposed to the analyte enriched nitrogen stream at an equable flow rate of 200 mL/min. To verify the response of the interrogation system, the analyte concentration was increased stepwise with intermediate washing of pure nitrogen. The respective change of the reflected wavelength was then recorded by the measurement system SIS:Lab2. The sensor was exposed to the naphthalene saturated nitrogen for 2 min and purged with pure nitrogen subsequently for 4 min to ensure the return of *λ_B_* to its reference level.

## 3. Results and Discussion

**Figure 3.**
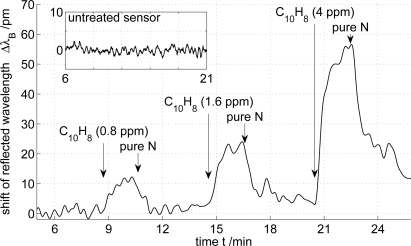
Shift of the reflected wavelength of the sensor coated with allylated *γ*-cyclodextrin to naphthalene in various concentrations (response of the untreated sensor is shown in the inset). Smoothed by Savitzky–Golay filtering.

The response of the sensors to a change in its immediate vicinity (penetration of the evanescent field into the surrounding media) is expressed in a shift of the intensity maxima of the reflected wavelength. Figure 3 shows the response of the sensor coated with octakis(2,3,6-tri-*O*-allyl)-*γ*-cyclodextrin to concentrations of 0.8 ppm, 1.6 ppm and 4 ppm of naphthalene in solvent vapor.

**Figure 4.**
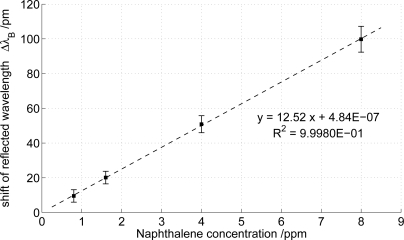
Relationship between the response intensity (average value of the converging signal) and the naphthalene concentration. The error bars take account of the standard deviation of the converging signal.

Figure 3 reveals that the observed trace shows a quasi-instantaneous response within the sampling rate of the interrogation system of 2 Hz upon naphthalene exposure. The total spectral shift of λ*_B_* was found to rise with increasing naphthalene concentration and fall with decreasing naphthalene concentration, respectively. The decay to almost the initial basic state manifests the reversibility of the sensor system. The behavior of the uncoated sensor is shown in the inset of Figure 3. Since the untreated sensor shows no reaction to equal naphthalene concentrations, the observed response of the functionalized sensor can be attributed to the interaction between the cyclodextrins and the analyte. In addition, no significant temperature-provoked cross-sensitivity was found. However, any monitored effect measured by the reference grating is taken into account in our data. The overall shift of λ*_B_* and its dependency to the analyte concentration is depicted in Figure 4.

The series in Figure 4 reveals the direct linear relationship of the overall wavelength shift to the amount of naphthalene concentration in the observed range. Therefore, the sensibility of the sensor system was found to be 12.5 ± 1.5 pm/ppm. Considering the spectral resolution of the interrogation system of 1 pm, this corresponds to acquisition steps of 80 ± 10 ppb. However, for a reliable evaluation of the analyte concentration according to the condition Δλ*_B_* ≥ 3 *· σ*, where the standard deviation of the interrogation systems background noise *σ* was found to be 2 pm, the minimum detectable naphthalene concentration is 0.48 ± 0.05 ppm.

## 4. Conclusions

A highly sensitive optical refractive index sensor based on a specifically functionalized planar Bragg grating has been demonstrated. The system was applied for the detection and measurement of aromatic hydrocarbons such as naphthalene. The interrogation system shows a quasi-instantaneous response and a linear dependence of the Bragg wavelength with naphthalene concentrations of 12.5 ± 1.5 pm/ppm, corresponding to a reliable minimum detection limit of 0.48 ± 0.05 ppm. The combination of an easy functionalization method, an easy handling of the sensor structure in addition to a high and reversible sensitivity makes this sensor system an ideal candidate for the identification and measurement of arene gases such as naphthalene.
